# Substance use, injection risk behaviors, and fentanyl-related overdose risk among a sample of PWID post-Hurricane Maria

**DOI:** 10.1186/s12954-022-00715-4

**Published:** 2022-11-24

**Authors:** Roberto Abadie, Manuel Cano, Patrick Habecker, Camila Gelpí-Acosta

**Affiliations:** 1 University of Nebraska-Lincoln, Lincoln, NE 68588 USA; 2 Arizona State University, Central Avenue 800, Phoenix, AZ 85004 USA; 3 LaGuardia Community College, City University of New York, 31-10 Thomson Avenue, Long Island City, NY 11101 USA

**Keywords:** Hurricane Maria, PWID, Rural Puerto Rico, Injection behaviors, Risk environment, Health disparities, Fentanyl, Overdose

## Abstract

**Background:**

While natural disasters like hurricanes are increasingly common, their long-term effects on people who inject drugs are not well understood. Although brief in duration, natural disasters can radically transform risk environments, increasing substance use and drug-related harms.

**Methods:**

Based on a study of people who inject drugs (PWID) and injection risk behaviors in rural Puerto Rico, the present study uses data from two different phases of the parent study. Data for 110 participants were collected from December 2015 to January 2017, soon before Hurricane Maria landed in September 2017; the 2019 phase, in the aftermath of the hurricane, included a total of 103 participants. The present study’s main analyses used data from 66 PWID who participated in both the pre-Maria and post-Maria interviews (66 individuals measured at two time points, for a total of 132 observations), using mixed-effects binomial logistic regression to examine recent overdose experiences pre- and post-Maria. A separate descriptive analysis included all 103 participants from the 2019 interview.

**Results:**

After Hurricane Maria, some declines in injection frequency were observed (the percentage of people reporting injecting monthly or less increased from 3.0% before Hurricane Maria to 22.7% after Hurricane Maria). However, fewer PWID reported using a new needle for most or all injections. In the pre-Maria interview, 10.6% of participants indicated they had experienced an overdose during the year of the interview and/or the calendar year prior, and this figure increased to 24.2% in the post-Maria interview. In the regression analysis, the odds of reporting an overdose during the interview year and/or calendar year prior were three times as high post-Maria, relative to pre-Maria (odds ratio 3.25, 95% confidence interval 1.06–9.97).

**Conclusion:**

Substance use patterns, injection risk behaviors, and overdose episodes and deaths differed after Hurricane Maria, relative to before the hurricane, yet it is unclear to what extent these changes also reflect the simultaneous arrival of fentanyl. In preparation for future natural disasters, it is imperative to strengthen the health infrastructure by enhancing access and curbing barriers to syringe services programs and medications for opioid use disorder, particularly in rural or underserved locations.

**Supplementary Information:**

The online version contains supplementary material available at 10.1186/s12954-022-00715-4.

## Introduction

In September 2017, Hurricane Maria, one of the worst natural disasters in the history of Puerto Rico [1], devastated the island’s infrastructure. Clinics offering medications for opioid use disorders (MOUD) closed, syringe services programs (SSP) were interrupted, and the social networks and everyday lives of people who inject drugs (PWID) were disrupted. None of these effects were felt more acutely than in the rural interior, where health services were already severely deficient [2, 3]. The majority of MOUD clinics are concentrated in big urban centers, particularly in metropolitan San Juan, which also centralizes other health infrastructures [4]. Along with economic crises, pandemics, wars, or other natural disasters, hurricanes are considered “big events” because they alter the micro and macro structures in which vulnerable populations live and make risk decisions that impact their health [5–8].

While natural disasters like hurricanes and floods are increasingly common due to global warming, the long-term effects of these big events on PWID are not well understood. Some studies have shown an increase in mortality rates following tropical cyclones in the U.S. [9], but the impact on the injection practices, risk behaviors, and overdose risk among PWID is less well understood.

Loss of material possessions and resources in an already disadvantaged population and an increase in environmental stressors and PTSD (posttraumatic stress disorder) are often associated with a rise in substance use [10–12]. Hurricanes, although brief in duration, can transform risk environments [13], which can have lasting effects on risk behaviors and health outcomes among PWID. A study with PWID conducted after Hurricane Sandy in New York City showed an increase in the sharing of injection equipment and injection with somebody outside of their regular social networks; these were accompanied by a rise in opioid withdrawal episodes. At the same time, the authors found a decrease in access to MOUD and a reduction in HIV (human immunodeficiency virus) medication taking [14].

Studies in the United States show the heightened overdose vulnerability of Puerto Ricans when compared to non-Hispanic White and non-Hispanic Black individuals [15–17]. These overdose disparities practically mirror the HIV and HCV disparities also affecting Puerto Rican PWID in the continental United States [4, 18–23]. These health disparities are often connected to poverty and “ghettoization” in the United States [23] and to a lack of culturally appropriate disease and overdose prevention interventions [2, 22]. Additionally, drug user stigma greatly compromises their accessing and/or remaining in treatment [24–26]. In addition, intergenerational injection drug use in this population, like in other minority groups [27], cements health disparities.

Paradoxically, while natural disasters might disrupt access to harm-reduction resources, these events do not seem to have lasting effects on drug availability. Dunlop et al. [28] find that after Hurricane Katrina hit New Orleans, the drug supply was briefly interrupted but quickly resumed, after drug dealers moved to other cities along with the displaced population [28–30]. These observations have been confirmed by other studies that showed that drug dealers arriving in new locations after Katrina were able to negotiate market access with already established dealers [31].

In order to contribute to the literature on post-disaster changes experienced by PWID, the present study examines drug overdose–related experiences in a sample of PWID in rural Puerto Rico before and after Hurricane Maria, a natural disaster that also coincided with the increase of illicitly manufactured fentanyl on the island [2]. To provide context to the overdose outcomes examined, the study also compares socioeconomic circumstances, drug use/injection practices, and treatment utilization before and after Hurricane Maria. While changes in injection behaviors and drug-related harms among PWID after natural disasters are generally assessed ex-post, as these events are unpredictable by their nature, the present study leverages epidemiological data collected before and after the hurricane. Rather than intending to determine whether any changes observed after Hurricane Maria were caused directly by the hurricane, the concurrent rise of illicitly manufactured fentanyl, or other factors, this study aims to elucidate post-hurricane shifts experienced by PWID in rural Puerto Rico, as these changes, regardless of cause, shape the risk environment for PWID. Through understanding the evolving needs of this high-risk group in the aftermath of Hurricane Maria and the era of fentanyl, we seek to enhance MOUD and SSP preparedness for future “big events”.

## Methods

### Data source and sample

The present study consists of a secondary analysis of data from two phases of a larger multi-phase study on social networks, HIV, and HCV (hepatitis C virus) among PWID in four rural areas of Puerto Rico (Cidra, Cayey, Comerío, and Aguas Buenas). In the multi-phase parent study, 315 participants were initially recruited via respondent driven sampling (RDS) for phase 1. Analysis of phase 1 RDS recruitment has been previously published, offering additional demographic and sociometric data about PWID in rural Puerto Rico before the arrival of Hurricane Maria [32, 33]. In phase 2 of the project, 33 participants from phase 1 were asked to complete extensive social network interviews, and their social connections were then in turn invited to participate in phase 2, resulting in 110 interviews. More information on the nature of the phase 2 interviews and resulting networks has been published [34]. All participants at phase 1 were at least 18 years old, alert at the time of the interview, and had injected drugs within the prior 30 days, as substantiated via track marks and completion of a questionnaire about injection practices. Track marks were identified by trained research assistants with extensive experience working with this population as syringe exchange providers. In this role, research staff were acquainted with the lesions and track marks left by intravenous drug use. In addition, staff conducted a training session where pictures of injection marks and non-injection marks were identified. Computer-assisted interviews were completed by trained field researchers, using a questionnaire adapted from the National HIV and Behavioral Surveillance (NHBS) Round 3 survey instrument [35]. All participants provided consent for the study and were compensated with up to $60 for participating in the survey and each study phase, including compensation for HIV/HCV tests. In addition, each RDS participant could receive $10 for each eligible participant brought to the study (with an upper limit of no more than three additional subjects per participant). Institutional Review Board (IRB) approval for the data collection in the parent study was provided by the University of Nebraska-Lincoln and the University of Puerto Rico.

The present study uses data from two different phases of the parent study: (1) the December 2015-January 2017 phase 2, hereafter labeled as the “pre-Maria (2016) interview,” and (2) a January-June 2019 phase, hereafter labeled as the “post-Maria (2019) interview.” Data for 110 participants were collected in the pre-Maria interview; the post-Maria interview included a total of 103 participants, 66 of whom had also participated in the pre-Maria (2016) interview. The present study’s main analyses used data from the 66 PWID who participated in both the pre-Maria (2016) and post-Maria (2019) interviews (66 individuals measured at two time points, for a total of 132 observations). A separate analysis included all 103 participants from the post-Maria (2019) interview. In the present study, listwise deletion was used for missing data, which ranged from 0.0 to 3.0% on individual variables examined.

### Measures

All measures included in the present study were based on participant self-report and were related to demographic characteristics, socioeconomic circumstances, drug use/injection behaviors, treatment utilization, and overdose. Demographic characteristics were assessed based on responses to the post-Maria (2019) interview, while both pre- and post-Maria responses were examined for socioeconomic circumstances, drug use/injection behaviors, treatment utilization, and overdose. Additional file 1: Table S1 details the timeframes (e.g., past year) for each variable in the pre- and post-Maria interviews.

*Demographic characteristics* included the area of residence (Cidra, Cayey, Comerío, Aguas Buenas, or other), age (in years), gender (man, woman, transgender), educational attainment (less than 12th grade, 12th grade or GED, beyond high school), marital status (single/never married, married/living as married, separated/divorced/widowed), birthplace (Puerto Rico, continental United States), and whether the participant had ever lived in the continental United States (yes/no). *Socioeconomic circumstances* included current homelessness (yes/no), monthly job income (none, $1–299, $300–699, $700+), current unemployment or disability (yes/no), current health insurance (yes/no), and inability to receive necessary medical care due to cost or access (yes/no).

*Drug use/injection practices* included age at first injection (less than 15 years old, 15–18, 19–24, 25 or older), injection frequency (monthly or less, less than daily but more than monthly, 1–3 times daily, 4–7 times daily, 8 or more times daily), new needle use for most or all injections (yes/no), use of a needle someone else had used, half of the time or more (yes/no), and sharing a cooker/cotton/water for at least half of all injections (yes/no). Daily injection (yes/no) was assessed for the following drugs: (a) heroin with cocaine, (b) xylazine with cocaine, (c) heroin alone, (d) powder cocaine alone, (e) crack cocaine, (f) buprenorphine, (g) xylazine alone, (h) methamphetamine, and (i) prescription opioids. Weekly non-injection drug use (yes/no) was assessed for (a) cannabis, (b) crack cocaine, (c) powder cocaine, (d) benzodiazepines, (e) prescription opioids, (f) heroin, (g) buprenorphine (from the street), (h) methamphetamine, (i) xylazine, (j) hallucinogens, and (k) ecstasy. Finally, weekly “binge drinking” (yes/no) was defined as more than five drinks (for males) or four drinks (for females) on one occasion, at least weekly.

Measures related to *treatment utilization* included participation in drug treatment of any kind in the past year (yes/no) and whether the participant attempted but was unable to enter drug treatment in the past year (yes/no).

The present study’s primary outcomes were *overdose-related* measures. In the parent study, overdose measures comprised cumulative totals/lifetime measures of the number of overdoses participants reported ever experiencing, rather than measures of overdoses within specific time frames (e.g., past-year/past-month overdose experiences). For the present study, therefore, we constructed a binary (yes/no) variable to capture whether each participant reported experiencing an overdose during the interview year and/or calendar year prior (that is, when assessed during the pre-Maria interview, whether each participant reported experiencing an overdose during 2015 and/or 2016, and, when assessed during the post-Maria interview, whether each participant reported experiencing an overdose during 2018 and/or 2019). This measure represents the closest approximation of a past-year overdose measure available within the existing data, although we chose to include overdoses in two calendar years (both the year of interview and the year before) for a more robust measure given our modest sample size. This variable was constructed based on participants’ responses to a question about the year in which their most recent overdose occurred, as assessed during both the pre-Maria and post-Maria interviews.

Three additional questions (lifetime measures) assessed overdoses within participants’ social networks: (a) the number of overdoses the participant reported ever witnessing, (b) the number of people the participant reported knowing who have experienced overdose, and (c) the number of people the participant reporting knowing who have died from a drug overdose. Finally, in the post-Maria (2019) interview only, participants also answered several questions regarding changes they had experienced after Hurricane Maria. Participants were asked for their perception of the frequency of overdoses among people who use drugs (a) during the time period *right after* Hurricane Maria, compared to before (options: fewer, same, more) and (b) *since* Hurricane Maria, relative to before (options: fewer, same, more). While the wording of the first question focused only on the period immediately after the hurricane occurred, the second question was intended to encompass a broader period of time, from the day of the hurricane to the day of the interview.

### Analyses

All analyses were completed in Stata/MP 16.1. First, descriptive statistics (for demographic measures and age at first injection) were calculated for the 66 PWID who had participated in both the pre-Maria (2016) and post-Maria (2019) interviews. Next, these 66 participants’ responses in the pre-Maria and post-Maria interviews were compared with respect to socioeconomic circumstances, drug use/injection practices, and treatment utilization. Due to the data’s paired responses (repeated measures for the same participants at two different time points), McNemar tests were used for binary variables, and the paired-samples sign test was used for ordinal variables. In consideration of the modest sample size, the mid-*p* version of the McNemar test [36] was chosen to maximize statistical power while utilizing a more conservative option than the classic McNemar test (limiting type I error) [36, 37].

Next, mixed-effects binomial logistic regression analysis was used to model time of interview (pre-Maria [2016] or post-Maria [2019]) as a predictor of the overdose-related outcome variable (a yes/no measure of whether the participant reported experiencing an overdose in the interview year and/or prior calendar year), with random intercepts at the participant level due to the non-independence of observations clustered within participants. Results were expressed as odds ratios (ORs) with 95% confidence intervals (CIs). Box plots, plotted with the user-written program *stripplot* [38], were then utilized to depict the raw distributions of the three other overdose-related variables: the reported number of overdoses witnessed, number of people known who had experienced overdose, and number of people known who had died of an overdose, as assessed in the pre-Maria (2016) and post-Maria (2019) interviews. Descriptive statistics (means and standard deviations [SDs]) were also calculated for each of these measures pre- and post-Maria. Finally, a column chart was used to depict the relative frequency of each response to the two post-Maria interview questions regarding participants’ perceptions of changes in the frequency of overdoses among people who use drugs.

## Results

### Sample characteristics

Of the 66 PWID who participated in both phases of data collection (pre-Maria and post-Maria interviews), 89.4% identified as men (10.6% women, 0% transgender), and ages ranged from 24 to 67 years, with a mean of 47.7 and standard deviation of 9.8 at the time of the post-Maria interview. Two of every three (66.7%) participants were single/never married, 15.1% were married or living as married, 15.1% were separated/divorced/widowed, and 3.0% did not provide a marital status. Approximately 43.9% reported completing less than a high school education, 33.3% had completed high school or received a General Education Development (GED) diploma, and 22.7% had attended education beyond high school. More than two in every five reported initiating injection drug use at age 18 or below (16.7% at below age 15 and 25.8% at ages 15–18), and one in three reported first injecting drugs at ages 19–24.

Nearly three in every four (72.7%) participants indicated that they had previously lived in the continental United States, although nearly all participants (97.0%) had been born in Puerto Rico. Most participants resided in Cidra (43.9%), followed by Comerío (22.7%), Cayey (16.7%), and Aguas Buenas (7.6%).

### Repeated measures for 66 participants before and after Hurricane Maria

#### Socioeconomic circumstances, drug use/injection practices, and treatment utilization

Table 1 details the socioeconomic circumstances, drug use/injection practices, and treatment utilization of 66 participants as assessed during the pre-Maria (2016) and post-Maria (2019) interviews. With respect to socioeconomic measures, participants’ monthly job income differed by interview time (*p* = 0.005). For example, during the pre-Maria interview, 18.5% of participants reported no monthly job income, and 27.7% reported earning between $1–299 monthly; during the post-Maria interview, 29.2% of participants reported no monthly job income, and 40.0% reported earning between $1–299 monthly.

**Table 1:** Socioeconomic circumstances, drug use/injection practices, and treatment utilization for 66 participants interviewed across two time points

	Pre-Maria (2016) interview, %	Post-Maria (2019) interview, %	*p* value
*Socioeconomic*			
Homelessness, current	10.9	12.5	0.754
Monthly job income, $			**0.005**
None	18.5	29.2	
1–299	27.7	40.0	
300–699	29.2	15.4	
700+	24.6	15.4	
Unemployed or disabled	81.8	71.2	**0.039**
Uninsured	13.6	16.7	0.581
Unable to receive needed medical care due to cost or access	16.7	6.1	0.057
*Drug use/injection practices*			
Injection frequency			**0.044**
Monthly or less	3.0	22.7	
Between daily and monthly	13.6	12.1	
1–3×/daily	40.9	31.8	
4–7×/daily	33.3	16.7	
8×/daily+	9.1	16.7	
Daily injection of			
Heroin with cocaine	68.2	54.6	0.064
Xylazine with cocaine	0.0	1.5	–
Heroin alone	15.4	21.5	0.227
Powder cocaine alone	7.6	7.6	–
Crack cocaine	1.5	0.0	–
Buprenorphine	1.5	0.0	–
New needle use, mostly or always	76.9	55.4	**0.008**
Use of needle someone else had used, half of the time or more	3.0	3.0	–
Sharing cooker/cotton/water, half of the time or more	53.9	30.8	**0.002**
Weekly non-injection use of:			
Cannabis	24.2	42.4	**0.007**
Crack cocaine	29.2	33.9	0.424
Powder cocaine	10.6	4.6	0.180
Benzodiazepines	25.8	42.4	**0.008**
Prescription opioids	9.1	7.6	0.754
Heroin	4.6	10.8	0.180
Buprenorphine (from the street)	7.6	10.6	0.549
Weekly “binge drinking”	19.7	24.2	0.454
*Treatment Utilization*			
Participated in drug treatment	43.9	50.0	0.383
Tried to enter treatment but unable	18.2	18.2	–

Daily injection of xylazine, methamphetamine, or prescription opioids and weekly non-injection use of methamphetamine, xylazine, hallucinogens, or ecstasy were not presented in the table due to 0 affirmative responses across both time periods. *p* values were calculated via McNemar mid-p tests for binary measures and paired-samples sign test for ordinal measures. Bold highlights *p* values below 0.05. *N* varies between variables, with a range of 64–66 participants for 128–132 observations total

Although no significant time differences were identified with respect to daily injection of any particular drug, overall injection frequency differed between the pre- and post-Maria interviews (*p* = 0.044). The observed proportion of participants who reported injecting with the lowest frequency (one time per month or less) was 3.0% pre-Maria yet 22.7% post-Maria, and the proportion who reported injecting 4–7 times daily was half as high post-Maria (16.7%) as pre-Maria (33.3%). A relatively low proportion (3.0%) of participants in the pre-Maria and post-Maria interviews reported using a needle someone else had already used for half or more of all injections. However, the proportion of participants reporting new needle use for most or all injections declined post-Maria (55.4%, compared to 76.9% pre-Maria; *p* = 0.008), although the proportion reporting sharing a cooker, cotton, or water for at least half of injections also declined (30.8% post-Maria, compared to 53.9% pre-Maria; *p* = 0.002). Finally, the proportion of participants who reported weekly noninjection use of cannabis or benzodiazepines increased from 24.2 to 25.8% pre-Maria, respectively, to 42.4% post-Maria (*p* = 0.007 and *p* = 0.008, respectively). No significant differences were observed for the treatment utilization measures examined.

### Overdose-related measures

During the pre-Maria interview, 10.6% of participants indicated that they had experienced an overdose during the interview year and/or the calendar year prior (that is, during 2015 or 2016). In contrast, during the post-Maria interview, 24.2% of participants reported an overdose during the interview year and/or the prior calendar year (that is, during 2018 or 2019). Table 2 provides results from the mixed-effects binomial logistic regression model used to examine differences in this outcome between the pre- and post-Maria interviews. The likelihood of reporting experiencing an overdose during the interview year and/or calendar year prior were more than three times as high in the post-Maria interview than the pre-Maria interview (OR, 3.25; 95% CI 1.06–9.97).

**Table 2:** Results of mixed-effects binomial logistic regression predicting self-reported overdose during interview year and/or year prior, by time of interview

	Experienced overdose during interview year and/or year prior, OR (95% CI)
Time of interview	
Pre-Maria (2016)	1.00 (reference)
Post-Maria (2019)	**3.25** (1.06–9.97)
*p* value	0.039

Data for 66 participants assessed at two time points (*n* = 132 observations). The regression model includes a random intercept at the participant level. Bold indicates significance at *p* < 0.05

*OR* odds ratio, *CI* confidence interval

**Fig. 1:**
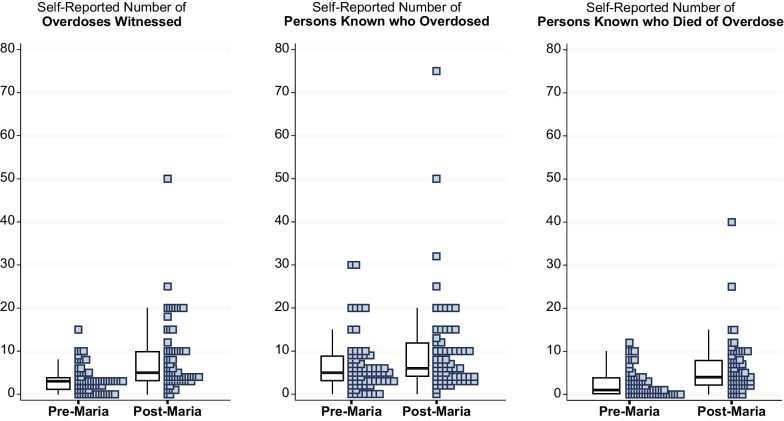
Distribution of responses for overdose-related outcomes assessed during pre-Maria (2016) and post-Maria (2019) interviews. *Note*. Each blue square represents one participant observation. In the boxplot, the box depicts the interquartile range and median, with a whisker 1.5 times the interquartile range

Figure 1 depicts the pre-Maria and post-Maria distributions of each of three lifetime measures related to overdoses participants reported within their social networks. Each blue square represents one participant response, the larger box represents the interquartile range, and the horizontal line within this box marks the median value. In the pre-Maria (2016) interview, the mean number of overdoses that participants reported witnessing was 3.18 (SD 3.11), and this figure was more than twice as high (8.20 [8.10]) in the post-Maria interview. The mean number of people that participants reported knowing who had experienced overdose was 6.65 (6.42) pre-Maria and 9.98 (11.59) post-Maria, while the mean number of people that participants reported knowing who had *died* of an overdose was 2.50 (2.96) in the pre-Maria interview and 5.98 (6.15) in the post-Maria interview.

### Post-Maria (2019) questions about perceptions of changes in overdose frequency

**Fig. 2:**
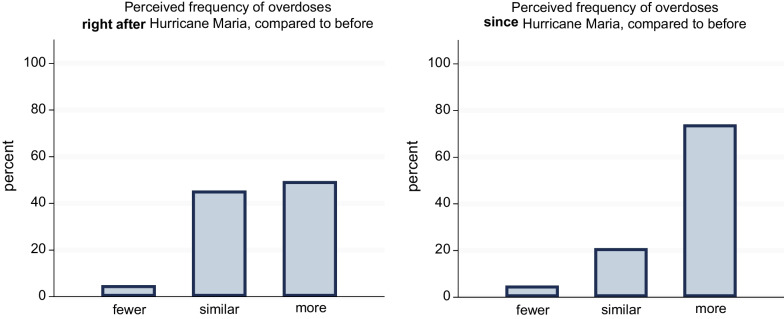
Participants’ reported perceptions of changes in overdose frequency among people who use drugs. *Notes*. Data from 103 participants interviewed in 2019

In the post-Maria (2019) interview only, all participants (*n* = 103) were asked about their perceptions of changes experienced after Hurricane Maria. As presented in Fig. 2, approximately half (49.5%) of participants indicated that they believed “more” overdoses occurred *right after* Hurricane Maria, and approximately three quarters (74.0%) of participants indicated they believed “more” overdoses had occurred in the time *since* Hurricane Maria, compared to before the hurricane.

## Discussion

Results of the present study document differences in substance use patterns, injection risk behaviors, and overdose experiences among PWID in rural Puerto Rico following Hurricane Maria. Although the study does not examine to what extent these changes resulted directly or indirectly from the hurricane or from the rise of fentanyl in drug supplies, the results nonetheless characterize an altered risk environment for PWID in Puerto Rico after Hurricane Maria, relative to before the hurricane. After Hurricane Maria, some declines in injection frequency were observed (the percentage of people reporting injecting monthly or less went from 3.0% pre-Maria to 22.7% post-Maria). However, at least one injection risk behavior seems to have increased, as fewer PWID reported using a new needle for most or all injections. This finding is in line with other studies evaluating the impact of “big events” on injection risk behaviors. For example, Aponte-Meléndez et al. [39] have shown that PWID in New York City had a higher rate of syringe reuse as well as sharing of injection equipment (i.e., cooker, cotton, and water) after the emergence of COVID-19 [39]. However, in our study, fewer PWID reported sharing injection equipment for at least half of their injections after Hurricane Maria. While this might suggest a reduction of a risk behavior, it might be also explained by a decrease in injection frequency and increase in noninjection use: the weekly use of cannabis and benzodiazepines increased from 24.2% and 25.8% pre-Maria to 42.4% for both post-Maria.

These changes in substance use and injection risks should not be entirely attributed to the occurrence of Hurricane Maria. Fentanyl, a synthetic opioid 50 times more powerful than morphine, and fentanyl-related analogs have been found in the opioid drug supply across the U.S. [40]. While epidemiological data about the presence of fentanyl in the drug supply on the island are lacking, this compound might have been present on the island before the hurricane, but according to media reports its use exploded after Hurricane Maria [41]. The arrival of fentanyl and its severe impacts may be reflected in the overdose outcomes among rural PWID in the study. The likelihood of reporting an overdose in the year of interview and/or the previous calendar year was three times as high in the post-Maria interview relative to the pre-Maria interview. Additionally, the number of overdoses witnessed and the number of people participants knew who had suffered an overdose event or died of an overdose increased notably. It is plausible, considering the exceedingly limited MOUD availability in this rural area (e.g., only one methadone clinic across four municipalities) and a post-Maria context in which clinics were not able to operate at full capacity and where transportation to these was significantly challenged (e.g., impassable roads for months, etc.), that the overdose outcomes in this study reflect this challenged drug treatment structure and the increased presence of fentanyl. In fact, findings on the right-after*-*Maria and since-Maria overdose-occurrence perceptions show a significant increase of the latter, suggesting an incremental fentanyl presence over time.

Overall, study findings show that overdose risk among rural PWID in the aftermath of Hurricane Maria has been particularly severe. We suggest that fentanyl-related overdose deaths and other drug-related harms among PWID in rural Puerto Rico constitute a syndemic event [2, 42] wherein the effects of Hurricane Maria, along with the arrival of fentanyl, an ongoing economic crisis, an overwhelmed MOUD infrastructure, and high rates of incarceration for drug-related offenses, all combined to exacerbate overdose death risks [43].

Studies have shown that while hurricanes like Katrina in New Orleans and Sandy in New York City increased injection risk behaviors, the disruption they caused in the drug markets was temporary. In Puerto Rico, Hurricane Maria seems to have had a more permanent—or syndemic—effect over drug supplies and overdose fatalities, considering the rising fentanyl presence in rural areas. With hurricanes and other natural disasters becoming more frequent and powerful, it is imperative to strengthen the health infrastructure, specifically by enhancing access and curbing barriers to SSP and MOUD, particularly in rural or underserved locations.

## Limitations

The self-reported measures used in this study are subject to response bias and recall bias. Moreover, the study was limited to measures available in the parent study, which represents a unique source of data from PWID in rural Puerto Rico before and after Hurricane Maria but was not designed to include all measures relevant for an analysis of pre- and post-hurricane conditions, as hurricanes are unforeseen events. In the absence of an available past-year measure of overdose, we assessed whether participants reported experiencing an overdose during the calendar year of the interview or the calendar year prior. The measures about overdoses witnessed and acquaintances who experienced or died from an overdose are cumulative/lifetime measures; as such, examining these measures pre- and post-Maria quantifies the increase in cumulative totals yet does not provide information about any possible changes in rates. These measures may also reflect the intertwined networks of participants, as multiple PWID may report the same witnessed overdose or the same acquaintance who experienced or died from an overdose. Furthermore, although the present study’s results are interpreted in light of the increase in fentanyl in Puerto Rico’s drug supply, the study was unable to examine fentanyl exposure directly, as questions about this emerging trend were not part of the original survey instrument utilized.

In the present study, 66 out of 110 participants (60%) from the pre-Maria interview participated in the post-Maria interview, and the sampling methodology used to engage this hidden and hard-to-reach population group precluded determination of the reasons that certain participants were not present in both interviews. Although 60% follow-up is toward the lower limits of traditional recommendations for cohort studies [44], the present study’s sample of 66 participants measured over two time periods should be viewed in consideration of the unique circumstances—a high-risk and hard-to-reach population and a massive natural disaster resulting in a substantial death toll and wave of outmigration. Finally, given the nonprobabilistic sample, it is unclear to what extent results may apply to PWID in rural Puerto Rico overall.

## Supplementary Information

**Additional file 1: Table S1.** Timeframes for the variables utilized in the study.
