# Correction: Substance use, injection risk behaviors, and fentanyl‑related overdose risk among a sample of PWID post‑Hurricane Maria

**DOI:** 10.1186/s12954-022-00724-3

**Published:** 2022-12-07

**Authors:** Roberto Abadie, Manuel Cano, Patrick Habecker, Camila Gelpí-Acosta

**Affiliations:** 1 University of Nebraska-Lincoln, Lincoln, NE 68588 USA; 2 Arizona State University, Central Avenue 800, Phoenix, AZ 85004 USA; 3 LaGuardia Community College, City University of New York, 31-10 Thomson Avenue, Long Island City, NY 11101 USA

**Correction to: Harm Reduction Journal (2022) 19:129** 10.1186/s12954-022-00715-4 Following publication of the original article [1], a text under Funding section has been inserted.

The original article has been corrected.

