# Supplementary material for: Substance use, injection risk behaviors, and fentanyl-related overdose risk among a sample of PWID post-Hurricane Maria
Source: Harm Reduct J. 2022 Nov 24;19:129. doi: 10.1186/s12954-022-00715-4 (PMC9694860; doi:10.1186/s12954-022-00715-4)
Supplement: Supplementary file 1 — Additional file 1: Table S1. Timeframes for the variables utilized in the study. [file 12954_2022_715_MOESM1_ESM.docx]

| **Additional file 1: Table S1**  *Timeframes for the variables utilized in the study* | | |
| --- | --- | --- |
|  | Timeframe for Measure | |
| Variable | 2016/2017 Interview | 2018/2019 Interview |
| **Sociodemographics** |  |  |
| Homelessness, current | Current | Current |
| Monthly income, $ | Current | Current |
| Unemployed or disabled | Current | Current |
| Uninsured | Current | Current |
| Unable to receive needed medical care due to cost or access | Past year or since previous interview | Past year |
| **Drug Use/Injection Practices** |  |  |
| Injection frequency | Past 3 months or since previous interview | Past year |
| Daily injection of _________^§^ | Past 3 months or since previous interview | Past year |
| New needle use | Past 3 months or since previous interview | Past year |
| Sharing cooker/cotton/water | Past 3 months or since previous interview | Past year |
| Weekly non-injection use of _______^§^ | Past 3 months or since previous interview | Past year |
| Weekly “binge drinking” | Past year or since previous interview | Past year |
| **Treatment Utilization** |  |  |
| Participated in drug treatment | Past year or since previous interview | Past year |
| Tried to enter treatment but unable | Past year or since previous interview | Past year |
| **Overdose-Related** |  |  |
| Number of overdoses experienced | Lifetime | Lifetime |
| Number of overdoses witnessed | Lifetime | Lifetime |
| Number of people known who overdosed | Lifetime | Lifetime |
| Number of people known who died from drug overdose | Lifetime | Lifetime |
| *Notes.* ^§^ Daily injection of… / Weekly non-injection use of… includes measures for any specific drug | | |
